# Principles to guide a regional agenda on the right to health

**DOI:** 10.1177/1468018115600123a

**Published:** 2015-12

**Authors:** Dainius Puras

**Affiliations:** UN Special Rapporteur on the right of everyone to the enjoyment of the highest attainable standard of physical and mental health; Vilnius University, Lithuania

Countries worldwide are striving to respect, protect and fulfil right to health and related human rights, but as we reach this critical juncture in the global development round, it is important that these rights are embedded in the SDGs and throughout the implementation process.

Let me first reflect on the consequences of treating human rights as divisible freedoms and entitlements. What are the implications of giving some groups of human rights preferential treatment over others, and of applying human rights principles to some people but not others?

During last two decades, convincing evidence has been accumulated on the negative impacts of inequalities and poverty on societal and individual health. Many examples presented by health and human rights movements illustrate the unacceptably high cost of inadequately addressing health as a social, economic and cultural right. When these rights are not protected (e.g. when neoliberal policies prevail) poverty, disempowerment, inequality and poor societal health become chronic, impacting on the well-being of large sections of the population who consequently suffer severely impaired health and life chances.

All too often, there remains an (erroneous) understanding that economic and social rights are subject to gradual realisation that they do not require immediate action and can wait until better circumstances or conditions are in place.

It is no coincidence that the outbreak of Ebola virus disease (EVD) epidemics happened in three countries of Western Africa having among the poorest standard of living and most fragile healthcare systems in the world. One of the lessons learned from this and other epidemic outbreaks is the importance of social medicine. Since the 19th century, social medicine has highlighted that many diseases and epidemics are in fact socially determined in their origin, and that effective primary prevention should properly address the causal factors.

Equally, there may be detrimental consequences to societal health when economic and social rights are properly addressed, but at the expense of civil and political rights. The unprecedented crisis of mortality and morbidity in the region of Central and Eastern Europe (CEE; Cornia and Paniccia, 2000) that has been going on since the beginning of 1990s illustrates this point. In a region of more than 20 countries and 400 million population, during the period of 1990–2000, there has been an ‘unplanned’ increase of 4 million deaths. Behind this is unhealthy response to the prolonged and unexpected psychosocial stress experienced by a large part of the population. The roots of this unhealthy response can be traced back 50 to 70 years ago, to the previous regime’s selective protection of social and economic rights while largely repressing civil and political rights of the population. Both prior to 1990 and afterwards, despite having relatively good health indicators compared to other regions (e.g. low rates of maternal infant and children under-5 mortality, high vaccination coverage, good access to primary and specialised care), large parts of population seem not have the skills and abilities to control their lives in a healthy way. In recent years, the poor health status of the population in the region has been defined by epidemics of destructive and self-destructive behaviour, including very high rates of suicides, other ‘external’ causes of deaths, and various forms of violence, or injuries (OECD, 2012).

Governments of the countries in the CEE region, with some notable exceptions, have failed to adequately respond to this social transition crisis. Even now, some 25 years after the onset of this crisis, in many countries medicalisation and institutionalisation are the predominant responses. Mental health services are to a large extent dependent on psychiatric hospitals, large long-term care institutions and overuse of psychotropic medication when epidemics of suicidal behaviour should be managed as a public health issue rather than as a psychiatric one. Many children, including the youngest (from birth to 3 years of age) are confined in large numbers in residential institutions in the CEE region (Brown, 2009), despite clear evidence that this is harmful to their development and well-being. This can be interpreted as a strongly embedded legacy of protecting the rights of children to survival and physical development, considered to be more important than the right to holistic development and well-being. Alternatives to institutionalisation, such as training of parents or primary care-givers and other psychosocial interventions to support families at the community level, all too often lose out to psychiatric and bio-medical interventions.

A commitment to human rights lies at the heart of the UN’s normative framework. Any hierarchy within human rights prioritising one right or one group of rights over another, or one population over others, has inevitable detrimental outcomes and leads to systemic violations of human rights. Selective approaches to human rights and fundamental freedoms undermine the crucial principle of meaningful participation and empowerment, which is of particular importance for the effective involvement of vulnerable and disadvantaged groups in decisions that affect them.

If the new SDGs are to stand any chance of being effectively realised, the commitment to human rights and the right to health must be a priority of first order. For this, the root causes of the global public health crisis must be tackled, and real improvements in the health of billions of people across all regions of the world are necessary.
